# Effects of a Ketogenic Diet Containing Medium-Chain Triglycerides and Endurance Training on Metabolic Enzyme Adaptations in Rat Skeletal Muscle

**DOI:** 10.3390/nu12051269

**Published:** 2020-04-30

**Authors:** Ayumi Fukazawa, Atsuko Koike, Takuya Karasawa, Momoko Tsutsui, Saki Kondo, Shin Terada

**Affiliations:** Department of Life Sciences, Graduate School of Arts and Sciences, The University of Tokyo, Tokyo 153-8902, Japan; koike.a5151@gmail.com (A.K.); karasawa3788@gmail.com (T.K.); momoko221917@gmail.com (M.T.); saki-kondo@g.ecc.u-tokyo.ac.jp (S.K.); terada@g.ecc.u-tokyo.ac.jp (S.T.)

**Keywords:** ketogenic diet, endurance training, MCT, β-hydroxybutyrate, OXCT, PDK4

## Abstract

Long-term intake of a ketogenic diet enhances utilization of ketone bodies, a particularly energy-efficient substrate, during exercise. However, physiological adaptation to an extremely low-carbohydrate diet has been shown to upregulate pyruvate dehydrogenase kinase 4 (PDK4, a negative regulator of glycolytic flux) content in skeletal muscle, resulting in impaired high-intensity exercise capacity. This study aimed to examine the effects of a long-term ketogenic diet containing medium-chain triglycerides (MCTs) on endurance training-induced adaptations in ketolytic and glycolytic enzymes of rat skeletal muscle. Male Sprague-Dawley rats were placed on either a standard diet (CON), a long-chain triglyceride-containing ketogenic diet (LKD), or an MCT-containing ketogenic diet (MKD). Half the rats in each group performed a 2-h swimming exercise, 5 days a week, for 8 weeks. Endurance training significantly increased 3-oxoacid CoA transferase (OXCT, a ketolytic enzyme) protein content in epitrochlearis muscle tissue, and MKD intake additively enhanced endurance training–induced increases in OXCT protein content. LKD consumption substantially increased muscle PDK4 protein level. However, such PDK4 increases were not observed in the MKD-fed rats. In conclusion, long-term intake of ketogenic diets containing MCTs may additively enhance endurance training–induced increases in ketolytic capacity in skeletal muscle without exerting inhibitory effects on carbohydrate metabolism.

## 1. Introduction

Endogenous carbohydrate stores are limited and thus sufficient to fuel only a few hours of continuous, submaximal (70–80% maximal oxygen uptake) exercise. Muscle and liver glycogen depletions are associated with the onset of fatigue and impairment of exercise performance [1]. Meanwhile, body fat deposits are large and represent a vast source of fuel for exercise. Therefore, enhancement of fat oxidation leads to glycogen sparing and has been suggested to improve endurance exercise performance [2]. It is well known that chronic endurance exercise training enhances the capacity for muscle and whole-body fat oxidation [3,4,5,6]. In addition, long-term intake of a high-fat diet additively enhances training-induced fat oxidation capacity [7,8,9]. In particular, a very high-fat and extremely low-carbohydrate diet, known as the ketogenic diet, enhances the capacity to convert fat to ketone bodies in the liver and utilize them in skeletal muscle [10,11]. Ketone bodies have been suggested to be more energy-efficient substrates than fatty acids [12,13,14]. Therefore, the ketogenic diet might be an effective dietary strategy to improve athletic performance in endurance events, especially in ultra-endurance events, such as ultra-marathon and triathlon races [15], and it has recently received much attention from athletes [16,17].

However, previous studies have demonstrated that extremely low-carbohydrate, very high-fat ketogenic diets induce substantial increases in pyruvate dehydrogenase kinase 4 (PDK4) content in skeletal muscle [18], which is a negative regulator of glycolytic flux [19]. Physiological adaptation to a ketogenic diet therefore diminishes carbohydrate utilization capacity during exercise [7,16,20]. In addition, ketogenic diets reduce muscle and liver glycogen levels as a consequence of their extremely limited carbohydrate content [21]. Thus, it has been suggested that low-carbohydrate ketogenic diets may not be suitable for athletes who engage in prolonged bouts of exercise involving high-intensity exertion, during which carbohydrates are utilized as a major energy substrate [22]. 

Medium-chain fatty acids (MCFAs), which consist of chains of 8–10 carbon atoms, have several unique properties in comparison with long-chain fatty acids (LCFAs). Unlike the majority of other dietary fats that are rich in LCFAs, medium-chain triglycerides (MCTs), which are composed exclusively of MCFAs, are hydrolyzed rapidly and the resultant MCFAs are absorbed directly by the liver through the portal vein [23,24]. MCFAs are more easily oxidized in the liver because their intramitochondrial transport does not require a carnitine palmitoyl transferase system [25], which is a rate-limiting step in mitochondrial β-oxidation. These characteristics make MCFAs a more ketogenic substrate than LCFAs in the liver. Therefore, incorporating MCTs into ketogenic diets, instead of long-chain triglycerides (LCTs), may allow for the consumption of more carbohydrate content and less fat content while preserving ketosis and enhancing ketone body utilization capacity without upregulating muscle PDK4 expression and thus inhibiting muscle carbohydrate metabolism. If MCTs have this effect, then MCT-containing ketogenic diets might be a valuable dietary strategy for athletes who require the ability to perform both high-intensity and endurance exercises.

The purpose of this study was therefore to examine the effects of long-term intake of a ketogenic diet containing MCTs and relatively more carbohydrate on endurance training–induced adaptations in metabolic enzymes in rat skeletal muscle tissue and to compare these effects with those observed under a conventional ketogenic diet composed exclusively of LCTs.

## 2. Materials and Methods

### 2.1. Animals and Diets

Seven-week-old male Sprague-Dawley rats (Japan SLC, Inc., Shizuoka, Japan) were individually housed in cages. The animal room was maintained at 23 ± 1 °C with 50 ± 5% humidity and illumination from 09:00 to 21:00. All animals were treated in accordance with national guidelines for the care and use of laboratory animals (Notification of the Prime Minister’s Office of Japan). The Animal Experimental Committee of The University of Tokyo approved all experimental protocols (approval no. 29–10).

During a 7-day acclimation period, the rats were allowed free access to water and a diet based on AIN-93 M formula. All animals were acclimated to the swimming exercise for 10 min per day for 4 days before being divided into the following three groups, matched for body weight and food efficiency. One group was continued on the AIN-93 M diet (CON group; *n* = 7), and the second and third groups were fed ketogenic diets containing LCTs (LKD; *n* = 7) or MCTs (MKD; *n* = 7), respectively. Table 1 and Table 2 show the compositions and fatty acid compositions, respectively, of each diet. Half the rats in each group performed a 2-h swimming exercise, 5 days a week, for 8 weeks (EX-CON; *n* = 7, EX-LKD; *n* = 7, EX-MKD; *n* = 7). Seven rats swam simultaneously in a barrel filled to a depth of 45 cm and with an average surface area of 230 cm^2^/rat. The temperature of the water was kept at 35 ± 1 °C during the swimming exercise.

All the rats were allowed ad libitum access to the experimental diets, and their food intake and body weight were recorded every second day. During the 8-week intervention, approximately 50-µL blood samples were collected from the tail vein using a heparinized microhematocrit capillary tube (Thermo Fisher Scientific, Waltham, MA, USA) to measure plasma β-hydroxybutyrate (βHB) and free fatty acid (FFA) levels at weeks 3, 4, 6, and 8. Collected blood samples were immediately separated into plasma and hematocrit by centrifugation (5 min at 10,000 rpm). Plasma samples were stored at −80 °C until analysis.

In a preliminary experiment, we confirmed that MKD elevated plasma βHB concentrations in sedentary rats to a level similar to that induced by LKD intake. Additionally, the group fed a large amount of MCTs was excluded from the present study because we found that intake of MCTs at levels equivalent to the intake of LCTs prescribed by the LKD treatment induced a remarkable reduction in food intake together with weight loss.

### 2.2. Tissue Sampling

Following the last training session (>20 h after the last bout of exercise), rats were sacrificed under anesthesia with isoflurane. The epitrochlearis and triceps muscles, which are mainly recruited during rat swimming exercises [26], and liver were quickly dissected out, promptly frozen in liquid N_2_, and stored at −80 °C until analysis. Intra-abdominal fat (the epididymal, mesenteric, and retroperitoneal fat pads combined) was removed and weighed. 

### 2.3. Analytical Procedure

#### 2.3.1. Energy Substrates in Plasma

Plasma βHB and free fatty acid (FFA) concentrations were respectively determined using Autokit 3-HB and NEFA-C test kits (Fujifilm Wako Pure Chemical Corporation, Osaka, Japan). The area under the curve (AUC) values for plasma βHB and free fatty acid concentrations above the baseline during the 8-week intervention were determined using the trapezoidal rule.

#### 2.3.2. Muscle and Liver Glycogen Concentration

For the measurements of the muscle and liver glycogen concentration, triceps muscle and liver were homogenized with 0.3 M perchloric acid. The glycogen concentrations were determined by the enzymatic methods described by Lowry and Passonneau [27] after acid hydrolysis.

#### 2.3.3. Muscle and Liver Homogenization

Frozen epitrochlearis muscle and liver tissues were homogenized in ice-cold radioimmunoprecipitation assay lysis buffer (EMD Millipore, Temecula, CA, USA) containing 50 mM Tris-HCl (pH 7.4), 150 mM NaCl, 0.25% deoxycholic acid, 1% NP-40, 1 mM ethylenediaminetetraacetic acid, and protease inhibitor cocktail (Sigma-Aldrich, St. Louis, MO, USA). The homogenates were frozen and thawed three times to disrupt intracellular organelles and rotated end-over-end at 4 °C for 90 min to solubilize the proteins. Homogenized samples were then centrifuged at 700× *g* for 5 min at 4 °C, and the supernatants were harvested.

#### 2.3.4. Western Blotting

Protein concentrations of the supernatants were measured with a bicinchoninic acid protein assay kit (Pierce, Rockford, IL, USA). Samples were prepared in Leammli sample buffer (Fujifilm Wako Pure Chemical Corporation) and heated 5 min in a heating block at 95 °C. Equal amounts of sample proteins were subjected to sodium dodecyl sulfate-polyacrylamide gel electrophoresis (with 10% or 12.5% resolving gels) and then transferred to polyvinylidene difluoride membranes at 200 mA for 90 min. After transfer, membranes were blocked for 1 h at room temperature in Tris-buffered saline (TBS) with 0.1% Tween 20 (TBS-T; 20 mM Tris base, 137 mM NaCl, pH 7.6) supplemented with 5% (*w*/*v*) bovine serum albumin. Membranes were incubated overnight with the following primary antibodies at concentrations of 1:500–1000 at 4 °C: anti-β-hydroxyacyl CoA dehydrogenase (βHAD, 1:500), anti- Succinyl-CoA:3-Oxoacid CoA-Transferase (OXCT, 1:1000), anti-Pyruvate dehydrogenase kinase 4 (PDK4, 1:1000) (ProteinTech Group, Chicago, IL), and anti-3-hydroxy-3-methylglutaryl-CoA synthase 2 (HMGCS2, 1:1000) (Abcam, Cambridge, MA, USA). After incubation with primary antibodies, membranes were incubated for 1 h at room temperature with secondary antibodies (goat anti-rabbit IgG, Jackson ImmunoResearch Laboratories, West Grove, PA, USA) diluted 1:5000 in TBS-T containing 1% nonfat powdered milk. Bands were visualized using an enhanced chemiluminescence reagent (GE Healthcare Life Science, Piscataway, NE, USA) and quantified by Image Studio Digits (Ver. 5.2; LI-COR Biosciences, Lincoln, NE, USA). The membranes were stained with Ponceau (Sigma-Aldrich) to verify equal protein loading across lanes. The intensities of immunobands were normalized to the total protein determined by quantifying all Ponceau red-stained bands in the relevant sample lane. We presented the date for protein contents as a fold expression relative to the mean values observed in the SED-CON group.

#### 2.3.5. Stripping and Reproving Membranes

The removal of primary and secondary antibodies from the membranes was achieved by incubating the membranes in stripping buffer (Sigma-Aldrich) at room temperature for 15 min. Then, membranes were washed for 10 min in TBS-T and blocked, as indicated above, for 1 h at room temperature. After that, membranes were ready to reprove with antibodies.

#### 2.3.6. Statistical Analysis

Data are presented as the mean ± standard error of the mean (SEM). Statistical analyses were performed using two-way analysis of variance (ANOVA) to examine the effects of diet and endurance exercise training. If significant interactions or main effects of diet or endurance exercise training were observed, the Tukey–Kramer multiple-comparison test was performed to examine the differences among groups. 

## 3. Results

### 3.1. Final Body Weight, Total Energy Intake, Intra-Abdominal Fat Weight and Food Efficiency

The EX group had significantly lower final body weight compared with the SED group. Moreover, a significant main effect of diet on the final body weight was observed and was associated with significantly lower body weight in the LKD and MKD groups compared with the CON group. However, there was no significant difference in the final body weight between the LKD and MKD groups (Table 3). 

The total energy intake was significantly lower in the EX group than in the SED group. A significant main effect of diet on total energy intake was observed, with the MKD group having significantly lower total energy intake than the CON and LKD groups (Table 3).

The total intra-abdominal fat weight in the EX group was significantly lower than that observed in the SED group. There was also a significant main effect of diet on total intra-abdominal fat weight. Total intra-abdominal fat weight was significantly lower in the MKD group compared with the CON and LKD groups (Table 3).

A significant interaction between diet and endurance exercise training for food efficiency was observed. Subsequent post hoc tests revealed that in both the SED and EX groups, the food efficiencies in the LKD and MKD groups were significantly lower than that observed in the CON group (Table 3).

### 3.2. Plasma β-Hydroxybutyrate and Free Fatty Acid Concentrations

**Figure 1:**
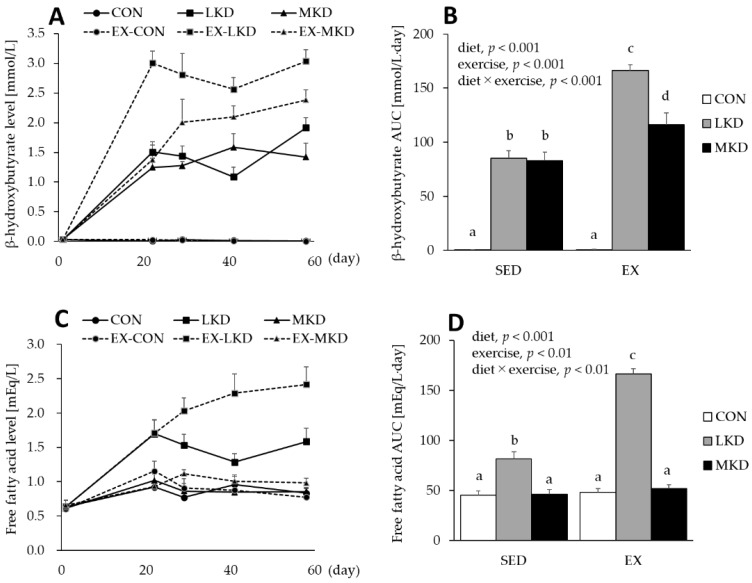
(**A**) Plasma β-hydroxybutyrate and (**C**) free fatty acid concentrations. The area under the curve (AUC) values for plasma (**B**) βHB and (**D**) FFA during the 8-week intervention were calculated in accordance with the trapezoidal rule. Two-way ANOVA was performed, followed by Tukey–Kramer multiple-comparison tests. Means labeled with the same letter are not significantly different from each other. Values are means ± SEM, *n* = 7.

As shown in Figure 1A, dietary intakes of LKD and MKD increased plasma βHB concentrations. A significant interaction between diet and training was observed for plasma βHB AUC during the 8-week intervention. Despite the lower lipid content of the diet, plasma βHB concentration in the SED-MKD group increased to a level similar to that attained in the SED-LKD group, and plasma βHB AUCs were significantly higher in the SED-MKD and LKD groups than the SED-CON group. Both the EX-LKD and EX-MKD groups also had significantly higher plasma βHB AUCs compared with the EX-CON group and all SED groups. However, the plasma βHB AUC in the EX-MKD group was significantly lower than that observed in the EX-LKD group (Figure 1B). 

A main effect of diet or endurance training on plasma FFA AUC during the 8-week intervention was observed. In addition, a significant interaction between diet and endurance exercise training was also observed for plasma FFA AUC. The LKD induced substantial increases in plasma FFA level (Figure 1C), and plasma FFA AUCs in both the SED- and EX-LKD groups were significantly higher than those observed in the respective CON and MKD groups. Moreover, plasma FFA AUC in the EX-LKD group was significantly higher than that in the SED-LKD group. In contrast, plasma FFA levels in both the CON and MKD groups were maintained at the pre-intervention basal level, whereas in both the SED- and EX-groups, there was no significant difference in plasma FFA AUC between the CON and MKD groups (Figure 1D).

### 3.3. HMGCS2 Protein Content in the Liver

**Figure 2:**
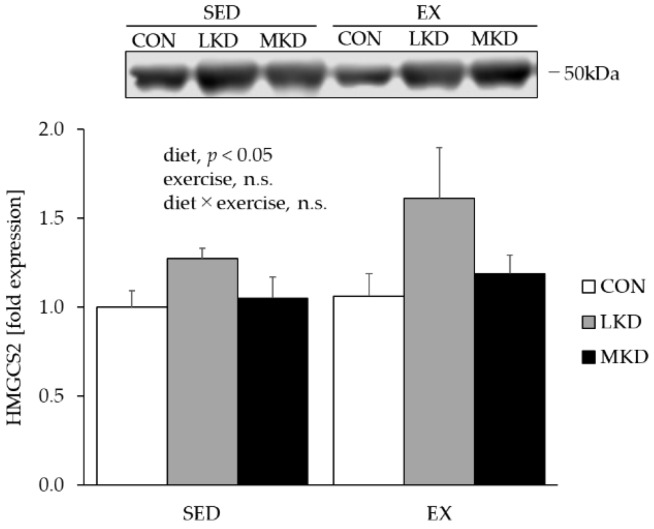
Protein expression of HMGCS2 in liver tissue. Two-way ANOVA was performed, followed by a Tukey–Kramer multiple-comparison test. Values are means ± SEM, *n* = 7: n.s., not significant. A representative western immunoblot image is shown.

Because the rate-limiting step of ketone body synthesis in the liver is the condensation of acetyl-CoA into HMG-CoA by mitochondrial HMGCS2 [28], we evaluated the hepatic HMGCS2 content as a marker of ketone body synthesis capacity (Figure 2). A significant main effect of diet on HMGCS2 protein content was observed and resulted in significantly higher HMGCS2 protein levels in the LKD group compared with the CON group. Moreover, HMGCS2 protein content tended to be higher in the MKD group than the CON group (*p* = 0.08). On the other hand, no significant difference in HMGCS2 protein level was observed between the LKD and MKD groups.

### 3.4. OXCT Protein Content in Epitrochlearis Muscle

As a small, polar molecule, βHB is utilized in extrahepatic tissues such as skeletal muscle, heart, and brain tissues. Once taken up by those tissues, βHB is converted to acetoacetyl-CoA for use as an energy substrate in mitochondria by OXCT, whose expression level is thought to reflect the ketolytic capacity of tissues [29]. 

**Figure 3:**
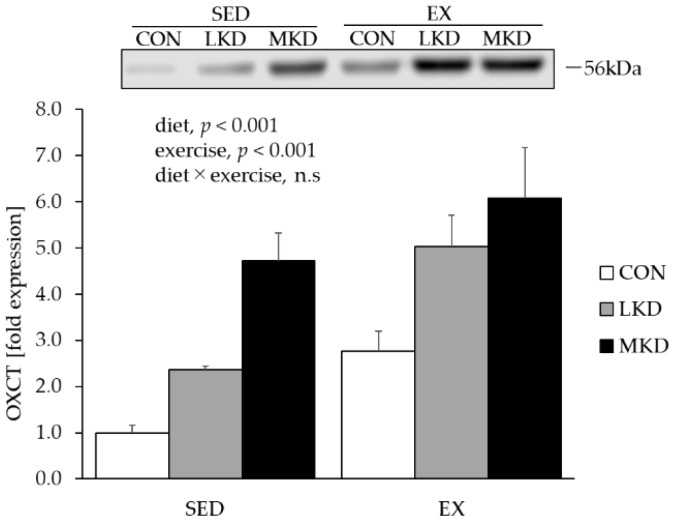
Protein expression of OXCT in epitrochlearis muscle tissue. Two-way ANOVA was performed, followed by a Tukey–Kramer multiple-comparison test. Values are means ± SEM, *n* = 7; n.s., not significant. A representative western immunoblot image is shown.

A main effect of endurance training on OXCT protein expression in epitrochlearis muscle tissue was observed, with the EX group having significantly higher expression compared with the SED group (Figure 3). Moreover, a significant main effect of diet on muscle OXCT expression was observed. The LKD group had significantly higher OXCT protein content in epitrochlearis muscle tissue than did the CON group. A further significant increase in muscle OXCT expression was observed in the MKD group, resulting in significantly higher OXCT content compared with the CON and LKD groups.

### 3.5. βHAD Protein Content in Epitrochlearis Muscle Tissue

To evaluate fatty acid oxidative capacity in skeletal muscle, we measured protein content of βHAD, which is a key enzyme in fatty acid β-oxidation and has been shown to be significantly associated with fatty acid oxidation rate during exercise [30].

**Figure 4:**
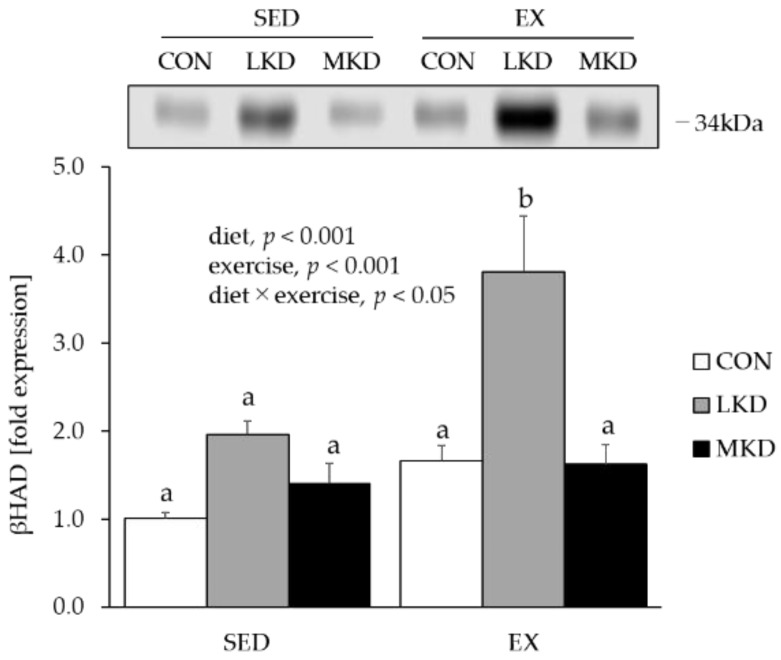
Protein expression of βHAD in epitrochlearis muscle tissue. Two-way ANOVA was performed, followed by a Tukey–Kramer multiple-comparison test. Means of treatments labeled with the same letter are not significantly different from each other. Values are means ± SEM, *n* = 7. A representative western immunoblot image is shown.

While a positive main effect of diet or endurance training on muscle βHAD protein content was observed, their interaction was also statistically significant (Figure 4). A subsequent post hoc test revealed that the protein expression level of βHAD in epitrochlearis muscle tissue of the EX-LKD group was significantly higher than those in the other groups.

### 3.6. PDK4 Protein Content in Epitrochlearis Muscle Tissue

Previous studies have demonstrated that extremely low-carbohydrate, very high-fat ketogenic diets reduce the capacity for glucose oxidation in skeletal muscle via upregulation of PDK4 content, which phosphorylates and inactivates the pyruvate dehydrogenase complex [18]. Therefore, we evaluated PDK4 protein content as a marker of negative regulation of glycolytic flux.

**Figure 5:**
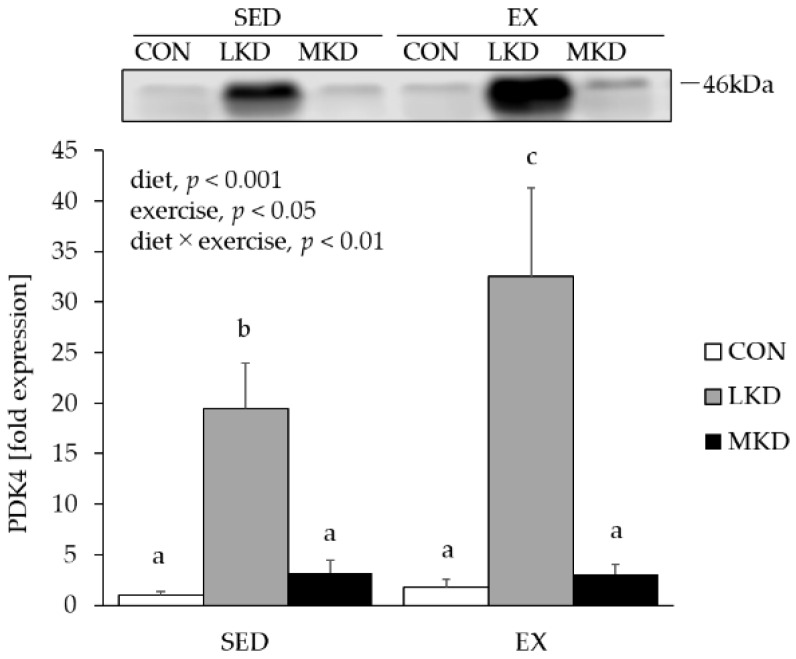
Protein expression of PDK4 in epitrochlearis muscle tissue. Two-way ANOVA was performed, followed by a Tukey–Kramer multiple-comparison test. Means of treatments labeled with the same letter are not significantly different from each other. Values are means ± SEM, *n* = 7. A representative western immunoblot image is shown.

A main effect of diet or endurance training on muscle PDK4 protein content was observed (Figure 5). Moreover, a significant interaction between diet and endurance exercise training was also observed for PDK4 protein content in epitrochlearis muscle tissue. In both the SED and EX groups, LKD intakes induced a substantial increase in PDK4 protein expression in epitrochlearis muscle tissue. Moreover, muscle PDK4 protein level in the EX-LKD group was significantly higher than that in the SED-LKD group. On the other hand, such an increase in PDK4 protein content was not observed in either the SED-MKD or EX-MKD groups.

### 3.7. Glycogen Concentrations in Triceps Muscle and Liver Tissues

**Figure 6:**
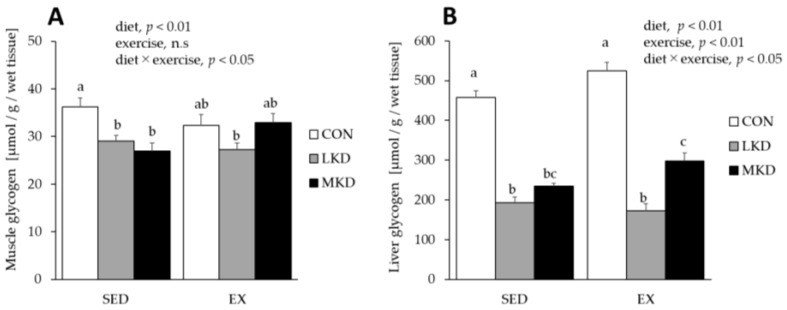
Glycogen levels in the triceps muscle tissue (**A**) and liver tissue (**B**). Two-way ANOVA was performed, followed by Tukey–Kramer multiple-comparison tests. Means of treatments labeled with the same letter are not significantly different from each other. Values are means ± SEM, *n* = 7.

A significant interaction between diet and endurance exercise training on muscle glycogen concentration was observed (Figure 6). Muscle glycogen concentration in the SED-LKD group was significantly lower than those observed in the SED-CON and -MKD groups. Although the differences did not reach statistical significance, the tissues from the EX-LKD group tended to have lower muscle glycogen concentrations compared with those from the EX-CON and -MKD groups. In contrast, although muscle glycogen concentration was significantly lower in the SED-MKD group than the SED-CON group, there was no significant difference between the EX-MKD and the EX-CON groups.

A significant interaction between diet and endurance exercise training was observed for liver glycogen concentration. In both the SED and EX groups, liver glycogen concentrations in the LKD and MKD groups were significantly lower than those in the CON group. However, liver glycogen concentration was significantly higher in the EX-MKD group than the EX-LKD group.

## 4. Discussion

Ketogenic diets are widely used as a weight loss strategy because they can potently suppress appetite and reduce energy intake [31,32]. Although, in the present investigation, the LKD-fed rats had similar total energy intake relative to the CON group rats, they showed significantly lower body weight and total intra-abdominal fat mass. As shown in Table 3, the LKD group had lower food efficiency, suggesting that LKD intake may prevent weight gain possibly through an increase in energy expenditure but not a decrease in energy intake. In the present investigation, we observed a further significant decrease in total intra-abdominal fat mass in the MKD group, especially in the EX-MKD group (the MKD intake and endurance training additively decreased total intra-abdominal fat mass) (Table 3). It has been well documented that MCT intake has body fat–lowering effects because it stimulates diet-induced thermogenesis and energy expenditure [33,34]. In addition, Ooyama et al. reported that MCT ingestion suppresses subsequent food intake in rats, possibly owing to an increase in hepatic ATP content [35]. Our results showing that the MKD group exhibited significantly lower total energy intake as well as lower food efficiency confirmed the findings of previous studies and provided additional evidence that the MKD is a more effective dietary strategy to control body weight and body fat.

In the SED group, LKD intake elevated plasma βHB concentration by up to ~1.5 mmol/L (Figure 1A). The LKD-induced ketosis might be mediated, at least in part, by higher expression of hepatic HMGCS2 (Figure 2), which is a rate-limiting factor in the synthesis of ketone bodies [28]. In contrast, plasma βHB concentration in the SED-MKD group increased to a level similar to that attained in the SED-LKD group without a significant increase in hepatic HMGCS2 expression (Figure 2). MCTs are absorbed via the portal vein and transported directly to the liver, where they are rapidly oxidized and converted to ketone bodies [23,24]. These unique properties made the MKD treatment more ketogenic, even though it was composed of more carbohydrates (18% of total energy vs. 1% in the LKD treatment) and did not upregulate hepatic HMGCS2 content.

Although both ketogenic diets in combination with endurance training induced further increases in plasma βHB concentration, the EX-MKD group showed significantly lower plasma βHB concentration compared with the EX-LKD group (Figure 1A). The lower plasma βHB concentration in the EX-MKD group might be caused by either diminished ketogenesis in the liver or enhanced ketolysis in other organs such as skeletal muscle. As shown in Figure 3, the MKD and endurance training treatments additively increased content of a key ketolytic enzyme, OXCT, in epitrochlearis muscle, resulting in the highest expression in the EX-MKD group. A previous study demonstrated a significant positive correlation between muscle OXCT content and ketone body utilization [29]. It is therefore more likely that the EX-MKD group could utilize more ketone bodies during training, accounting for their lower plasma βHB concentration compared with the EX-LKD group. 

Oxidation of ketone bodies (βHB) yields more ATP per mole of substrate compared with oxidation of the end-glycolytic substrate pyruvate [36]. In addition, ketone bodies increase the free energy released from ATP hydrolysis by reducing the mitochondrial NAD couple and oxidizing the coenzyme Q couple, thereby increasing the redox span between complex I and complex II of the mitochondrial electron transport chain [37]. These energetic characteristics of ketone bodies enabled a working, perfused rat heart to increase the efficiency of hydraulic work by ~30% compared with pyruvate [12]. Based on these findings, ketone bodies are therefore thought to be the most energy-efficient fuel. The MKD treatment, which additively enhanced the endurance training–induced increase in muscle ketolytic capacity, may therefore be a beneficial dietary intervention to improve endurance exercise performance. Previous studies demonstrated the lower adherence to a conventional ketogenic diet, because it consists exclusively of fat with an extremely limited carbohydrate content [38]. In contrast, the MKD treatment employed in this study could induce beneficial metabolic adaptations, as mentioned above, despite there being a relatively higher carbohydrate content (~18% energy from carbohydrates) and therefore might be a more feasible dietary intervention compared with conventional ketogenic diets containing LCTs. 

Endurance exercise training and a high-fat diet intake independently and additively increased the protein content of mitochondrial fatty acid oxidation enzymes and fat oxidation capacity in skeletal muscle [39,40]. The high-fat diet-induced upregulation of muscle fatty acid oxidation enzymes is thought to be a result of the activation of a nuclear receptor, peroxisome proliferator-activated receptor (PPAR) β, by elevated blood FFA [41]. In accordance with the previous findings [42,43], the LKD treatment substantially elevated plasma FFA levels with concomitant increases in muscle βHAD protein content, with the EX-LKD group having the highest values (Figure 1D and Figure 4). In contrast, the MKD groups did not show such increases in muscle βHAD protein content, suggesting that the MKD treatment enhanced utilization capacity only for ketone bodies, but not fatty acids. Consistent with a previous study showing that even larger amounts of MCT failed to produce detectable increases in plasma FFA [44], the MKD-fed groups had lower plasma FFA levels, which led to lower muscle βHAD protein content. Because βHAD is a key enzyme in fatty acid β-oxidation and its activity has been shown to be significantly corelated with fatty acid oxidation rate during exercise [30], the long-term intake of LKD, but not MKD, in combination with endurance training might be a sufficiently strong stimulus to enhance muscle fatty acid utilization capacity. However, although fatty acids are important substrates during endurance exercise, metabolism of fatty acids leads to a reduction in mitochondrial NAD and mitochondrial coenzyme Q, thus causing a decrease in the free energy released from ATP hydrolysis and thereby requiring more oxygen [45]. Actually, Burke et al. demonstrated that adaptation to a conventional low-carbohydrate, high-fat ketogenic diet impaired exercise economy (causing higher oxygen consumption at the same exercise intensity) and negated the performance benefit of intensified training in elite race walkers [7]. Thus, athletes may derive little benefit from conventional ketogenic diets consisting of LCTs. 

A major concern associated with ketogenic diet intake in athletes is its effects on carbohydrate metabolism in skeletal muscle [22]. Numerous studies have shown that very low-carbohydrate, ketogenic diets reduce glycolytic enzyme activity and thus diminish carbohydrate oxidation in humans and animals [7,16,18,46], suggesting that ketogenic diets may impair high-intensity exercise performance, during which carbohydrates are utilized as major energy substrates. Potential mechanisms by which ketogenic diets induced inhibition of glycolytic flux is postulated to be upregulation of PDK4 expression in skeletal muscle [47]. Consistent with previous finding [18], we observed a huge increase in PDK4 protein content in epitrochlearis muscle tissue of the LKD-fed rats, in particular in the EX-LKD group (Figure 5). This result supports the notion that this type of ketogenic diet exerts deteriorating effects on muscle carbohydrate utilization and high-intensity exercise capacity. Because expression of PDK4 as well as βHAD are mediated by PPARβ [48], the higher plasma FFA levels in the LKD groups might be responsible for the LKD-induced muscle PDK4 expression via activation of PPARβ. In contrast, as shown in Figure 5, the MKD treatment completely prevented such an increase in muscle PDK4 protein content concomitant with lower plasma FFA levels, although it contained relatively higher amounts of fat and induced ketosis. Our results therefore suggest that long-term intake of ketogenic diets containing MCTs and relatively more carbohydrate may enhance the utilization capacity of ketone bodies, which are particularly energy-efficient substrates, in skeletal muscle tissue without exerting inhibitory effects on muscle carbohydrate metabolism.

Previous studies have reported that muscle and liver glycogen concentrations were lower following consumption of a low-carbohydrate, ketogenic diet [49,50], which may be another contributing factor to the ketogenic diet-induced impairment in high-intensity endurance exercise performance. In this study, as a consequence of their lower carbohydrate intake, both ketogenic diet groups had significantly lower muscle and liver glycogen levels (Figure 6). However, the MKD-fed rats had less reduction in muscle and liver glycogen concentration than did the LKD-fed rats (Figure 6). These unique characteristics of the MKD treatment, which have reduced inhibitory effects on glycogen content as well as glycolytic enzyme levels, make this diet suitable for athletes who need the ability to perform prolonged high-intensity exercise that relies heavily on energy coming from glycolytic pathways.

This study has several limitations. First, we unfortunately could not measure other blood energy substrates such as plasma glucose and evaluate exercise capacity in humans or in rats. Thus, it remains unclear whether MKD treatments have athletic performance–enhancing effects or not, especially in prolonged high-intensity exercise. Second, this study was performed on only male rats. The results obtained in this study may not be directly extrapolated to female rats as well. Finally, we did not evaluate the effect of intake of LKD containing more carbohydrate at levels equivalent to the MKD in this study. We therefore could not rule out the possibility that consumption of relatively more carbohydrate rather than MCTs intake was responsible for the MKD-induced adaptations. Future extensive studies are required to solve these issues.

## 5. Conclusions

Long-term intake of a ketogenic diet containing MCTs and relatively more carbohydrate may reduce body weight and intra-abdominal fat mass and additively enhance endurance training-induced ketolytic capacity in skeletal muscle of male rats without exerting inhibitory effects on carbohydrate metabolism.

## Figures and Tables

**Table 1:** Compositions of the experimental diets.

Ingredient	(g/kg Diet)		
	CON	LKD	MKD
Cornstarch	520.80	−	189.00
Casein (>85% protein)	206.60	174.00	174.00
Sucrose	101.80	−	−
LCT	71.30	539.00	35.10
MCT	−	−	315.25
Cellulose fiber	51.00	238.00	238.00
Mineral mix (AIN-93-M-MX)	35.00	35.00	35.00
Vitamin mix (AIN-93-VX)	10.00	10.00	10.00
l-Cystine	2.50	2.50	2.50
Choline bitartrate (41.1% choline)	1.80	1.80	1.80
*tert*-Butylhydroquinone	0.08	0.08	0.08
Energy density (kcal/g)	4.00	5.60	4.50
Protein:Fat:Carbohydrate Ratio (% total energy)	21:16:63	12:87:1	16:66:18

CON, control group; LKD, ketogenic diet containing long-chain triglycerides (LCTs); MKD, ketogenic diet containing medium-chain triglycerides (MCTs).

**Table 2:** Fatty acid compositions of the experimental diets.

Fatty Acid	(g/100 g Fatty Acids)	
	CON and LKD	MKD
C8:0 *	ND	64.7
C10:0	ND	23.7
C16:0	10.6	1.2
C18:0	4.0	0.5
C18:1	24.9	2.9
C18:2	52.1	6.1
C18:3	6.7	0.8
C20:0	0.4	ND
C20:1	0.2	ND
C22:0	0.4	ND
C24:0	0.2	ND
Others	0.6	ND

* Number of carbon atoms: number of double bands; ND, not detected; CON, control group; LKD, ketogenic diet containing LCTs; MKD, ketogenic diet containing MCTs.

**Table 3:** Body weight, total energy intake, intra-abdominal fat weight, and food efficiency in rats.

											*p*-Values		
		CON			LKD			MKD			Diet	Exercise	Interaction
Initial body weight (g)	SED	274	±	7	274	±	4	274	±	4	n.s.	n.s.	n.s.
	EX	273	±	5	273	±	4	273	±	3			
Final body weight (g)	SED	518	±	21	478	±	16	432	±	8	*p* < 0.001 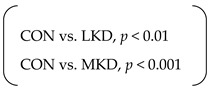	*p* < 0.001	n.s.
	EX	427	±	10	367	±	15	380	±	5			
Total energy intake (kcal)	SED	6754	±	269	6863	±	256	6151	±	126	*p* < 0.01 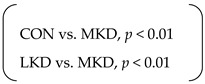	*p* < 0.001	n.s.
	EX	5969	±	133	5714	±	226	5135	±	42			
Intra-abdominal fat weight (g)	SED	40	±	3	36	±	2	24	±	2	*p* < 0.001 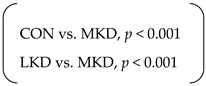	*p* < 0.001	n.s.
	EX	21	±	1	18	±	1	13	±	1			
Food efficiency (Δg/1000 kcal) *	SED	36	±	1 ^a^	30	±	1 ^b^	26	±	1 ^c^	*p* < 0.001 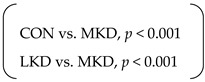	*p* < 0.001	*p* < 0.001
	EX	26	±	1 ^c^	16	±	1 ^d^	21	±	1 ^e^			

Two-way analysis of variance (ANOVA) was performed, followed by Tukey–Kramer multiple-comparison tests. Means labeled with the same letter are not significantly different from each other. Values are means ± SEM, *n* = 7. SED, sedentary; EX, exercise; n.s., not significant. * body weight gain divided by total energy intake (1000 kcal).
